# Coronary Anomaly and Coronary Artery Fistula as Cause of Angina Pectoris with Literature Review

**DOI:** 10.1155/2011/486187

**Published:** 2011-12-11

**Authors:** Jayanth Koneru, Anish Samuel, Meherwan Joshi, Aiman Hamden, Fayez E. Shamoon, Mahesh Bikkina

**Affiliations:** ^1^Seton Hall Cardiology Fellowship Program, St. Joseph Regional Medical Center, Seton Hall University 703 Main Street, Paterson, NJ 07503, USA; ^2^Seton Hall Cardiology Fellowship Program, St. Micheal's Medical Center, 111 Central Avenue, Newark, NJ 07102, USA

## Abstract

Coronary artery fistulas are rare anomalies of the coronary arteries that may sometimes cause symptoms by shunting blood flow away from the myocardial capillary network. We report the case of a 46-year old lady which shows the right coronary cusp giving rise to left main coronary artery called anomalous origin of a coronary artery (AOCA), and also a fistula between the left coronary artery and pulmonary artery. We describe our diagnostic approach and review the literature on the epidemiology, pathophysiology, the diagnostic modalities, and treatment options.

## 1. Introduction

Coronary artery fistula (CAF) is defined as an anomalous connection between a coronary artery and a major vessel or a cardiac chamber. It is an uncommon form of congenital heart disease. Most of the coronary anomalies are incidental findings during angiographic evaluation for coronary vascular disorders. Majority of these fistulas arise from the left anterior descending artery or from the right coronary artery [1]. Most of these patients are asymptomatic, but heart failure, angina, myocardial infarction, coronary steal, endocarditis, and dyspnea have been reported in some cases [2]. The management is complicated, and recommendations are based on anecdotal cases of very small retrospective series. We present a case of CAF with coronary anomaly. 

## 2. Epidemiology

The incidence of coronary anomalies varies between 0.6% and 1.5% of patients undergoing invasive cardiovascular imaging [3, 4]. CAF was first described in 1841, and it is defined as an anomalous connection between a coronary artery and a major vessel or cardiac chamber [5]. Its incidence in the general population is about 0.002%. The incidence of coronary pulmonary fistulas was similarly reported only to be 0.1% in a study of 11, 000 patients undergoing cardiac catheterization [6].

## 3. Anatomy

**Figure 1:**
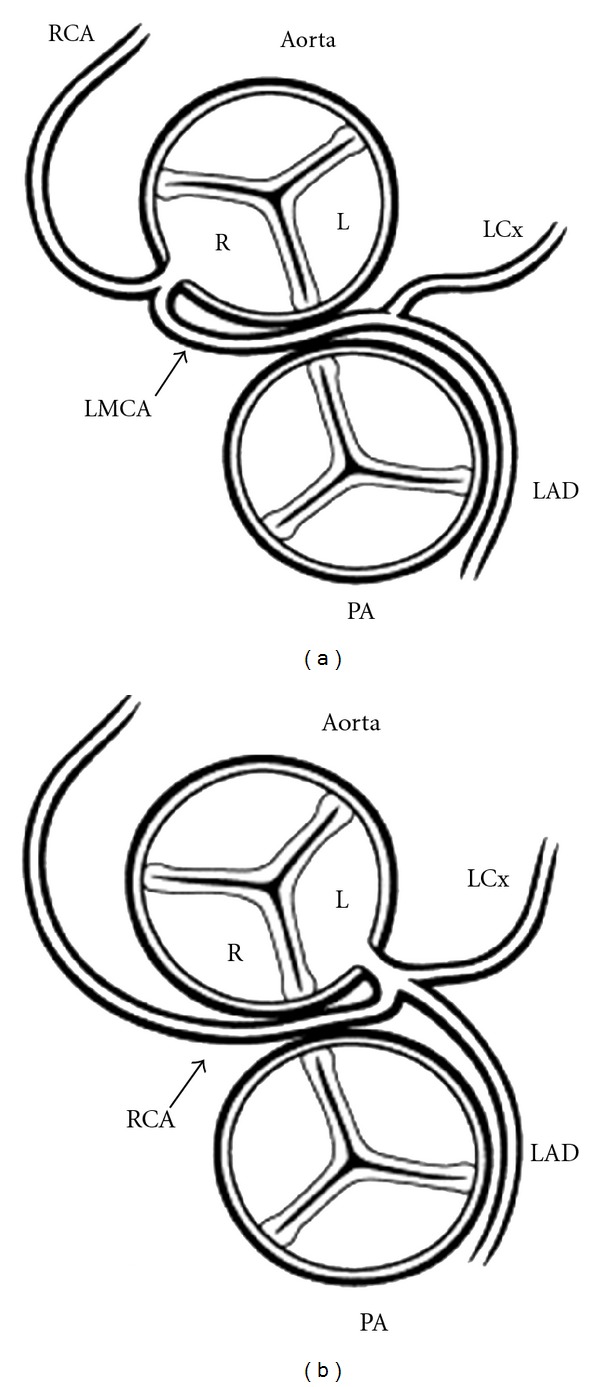
Schematic diagram of the two forms of anomalous origin of a coronary from the wrong sinus. Those are associated with myocardial ischemia. (a) Anomalous origin of the left coronary artery (LCA) from the right sinus of Valsalva. With anomalous origin of the left coronary, the left main coronary artery (LMCA) arises from the right aortic sinus (R) and passes between the great arteries before dividing into its two usual branches, the left anterior descending (LAD) and left circumflex (LCx) coronary arteries. (b) Anomalous origin of the right coronary artery (RCA) from the left sinus of Valsalva. With anomalous origin of the right coronary, the right coronary artery (RCA) arises from the left aortic sinus (L) and passes between the great arteries before coursing in its usual distribution. [26]. In each case, the anomalous coronary can be seen coursing between the aorta and pulmonary artery (PA).

AOCA arising from the opposite sinus of Valsalva (either the right coronary artery arising from the left sinus or the left coronary artery arising from the right sinus of Valsalva) especially an anomalous coronary artery coursing between the great arteries has been associated with myocardial ischemia, ventricular arrhythmias, and sudden death, (Figure 1) [7–26]. Two forms of AOCA not associated with myocardial ischemia or sudden death are AOCA from the noncoronary or posterior sinus of Valsalva [9, 10, 26]. The interarterial course of anomalous coronary artery (ACA) can be between the myocardial sulcus and the great arteries (intramyocardial) [15, 26] or within the anterior wall of the aorta between the great arteries (intramural) [7, 26]. 

The exact mechanisms that lead to myocardial ischemia in the patients with AOCA from the opposite sinus that course between the pulmonary and aortic roots are unclear, but several theories have been proposed. The anomalous coronary artery usually arises at an acute angle from the aorta, rather than perpendicularly; this may alter flow patterns into that coronary artery bed [15, 26]. Another proposed mechanism is compromised flow reserve secondary to slit like ostium associated with ACA [9, 26]. Finally, it was hypothesized that the interarterial course places the anomalous coronary at risk of compression between the great arteries. The ischemia could be from deformation of the anomalous coronary within the aortic wall during periods of systemic hypertension, particularly in patients who have an intramural course [26]. Because wall tension is determined by the radius of a vessel, the aorta will have greater wall tension than the intramural coronary within the aortic wall which results in deformation of the coronary and diminished cross-sectional area. During exercise, aortic wall tension increases, and with increasing aortic pressure the anomalous coronary becomes flattened and coronary reserve is reduced to a point where myocardial oxygen requirements are not met.

## 4. Pathophysiology

When the fistula drains into the right side of the heart, the volume load is increased to the right heart as well as to the pulmonary vascular bed, the left atrium, and left ventricle. When the fistula drains into the left atrium or the left ventricle, there is volume overloading of these chambers but no increase in the pulmonary blood flow. This results in varied appearance of dilation of different cardiac chambers due to the shunts on echocardiography. The size of the shunt is determined by the size of the fistula and the pressure difference between the coronary artery and the chamber into which the fistula drains. Occasionally, congestive cardiac failure occurs, and vey rarely, in adults, myocardial ischemia may occur [27, 28].

## 5. Case Report

A 46-year old, lady with past medical history significant for dyslipidemia and gastroesophageal reflux presents to her primary care physician with complaint of chest pain. Pain is described as 8/10, retrosternal, compressive pain, radiating to her neck bilaterally, intermittent in nature, and lasting for nine months. Patient mainly complained of pain during the day, while working, and had occasional relief when she was resting. Pain was associated with palpitations and shortness of breath and had an average of 2-3 episodes per week lasting 10–15 minutes each. No other symptoms were reported at that time. Patient denied having any family history of coronary artery disease or history of alcohol, tobacco, and illicit drug usage. Patient was proceeded to get an EKG and stress test. EKG did not show any acute abnormalities and Thallium stress test showed a mild degree of myocardial ischemia involving basal region of anterior wall, which was reversible. A cardiac catheterization was done, and it revealed left main coronary artery arising from the right cusp (Figure 1(a)) and a fistula between the left coronary and pulmonary artery (Figure 1(b)). Cardiothoracic surgery was consulted, and a ligation of the coronary artery fistula was done. On reevaluation of the patient 3 months after procedure, patient remains chest-pain-free and continues followup at the cardiology clinic.

## 6. Coronary Artery Fistulas

Congenital coronary artery fistula (CAF) is a rare, isolated anomaly of the coronary artery system that is defined as a direct communication between a coronary artery and another vascular structure. The involved coronary usually arises normally from the aorta and connects to one of the intracardiac chambers, systemic veins, or pulmonary artery by way of a tortuous anomalous branch. Fistulas more frequently involve the right coronary artery and usually drain into one of the right heart chambers [29]. Symptoms and signs are dependent on the size of the fistulous connection; rarely, large fistulas can have a significant left-to-right shunt with resultant congestive heart failure and cardiomegaly in infancy [30]. Most patients who have CAF are asymptomatic in childhood and present because of a continuous murmur that is appreciated along the precordium. This murmur can sound similar to a patent ductus arteriosus, although its parasternal location frequently suggests a different etiology. Late complications have been described in older adults and include bacterial endocarditis, congestive heart failure, and angina [29, 31, 32]. Two-dimensional and color Doppler echocardiography usually are diagnostic and can identify the involved coronary and its site of drainage [33]. Surgical closure is safe and effective [34, 35]. Recent advances in interventional techniques now allow closure of CAF in the cardiac catheterization laboratory with the use of detachable coils and balloons; this has become the initial treatment of choice in children and adults [36–38]. Closure of the fistula is indicated when symptoms are present; the need for prophylactic closure in asymptomatic children to prevent late complications remains controversial.

## 7. Diagnostic Evaluation

Coronary angiography remains the gold standard for diagnosis. Angiography helps define the artery of origin, recipient vessel, or chamber and the site of communication. Noninvasive techniques such as transthoracic echocardiography, transesophageal echocardiography, and magnetic resonance imaging are becoming increasingly popular for diagnosis and followup of CAFs. Combined two-dimensional and pulsed Doppler echocardiography demonstrates a dilated coronary artery, turbulent flow in the fistula and the recipient chamber [27, 28]. Transthoracic color Doppler echocardiography with a high-frequency transducer and a low Nyquist limit allows multiple coronary arteries—left ventricular microfistulae to be visualized. Transesophageal echocardiography is used intraoperatively to identify the precise location of the site of drainage of the fistula, which could not be accurately revealed with preoperative coronary arteriography [39]. Magnetic resonance imaging and multidetector computed tomography have also become the alternative methods to evaluate the anatomy, flow, and function of CAF [27, 28, 40, 41].

## 8. Management

The natural history of coronary artery fistulas is variable. Spontaneous closure secondary to spontaneous thrombosis, although uncommon, has been reported [42, 43]. The management is controversial, and recommendations are based on anecdotal cases or small retrospective series [44]. Antiplatelet therapy is recommended, especially in patients with distal coronary artery fistulas and abnormally dilated coronary arteries [44]. Prophylactic precautions against subacute bacterial endocarditis are recommended, as bacterial endocarditis is a known complication.

The main indications for closure are clinical symptoms especially of heart failure and myocardial ischemia and in asymptomatic patients with high-flow shunting, to prevent occurrence of symptoms or complications, especially in pediatric population [45]. Surgery and direct epicardial or endocardial ligations were traditionally viewed as the main therapeutic method for the closure of coronary artery fistulas [45]. Surgical correction is safe and effective, with good results [34, 45, 46]. Treatment of adult asymptomatic patients with nonsignificant shunting is still a matter of debate.

Catheter-based closure of the fistulous connection is the nonsurgical treatment option for closure of coronary fistulas, with good reported success [47–50]. Since its introduction in the early 1980s, transcatheter closure of coronary artery fistulas is now widely available and for nearly a decade is being recognized as an effective and safe treatment option for coronary artery fistulas [50]. Catheter closure can be performed with a variety of techniques, including detachable balloons, stainless steel coils, controlled-release coils, controlled-release patent ductus arteriosus coils, patent ductus arteriosus plug, regular and covered stents, and various chemicals [46–52]. The vast majority of the fistulas treated with catheter intervention were occluded with coils. Controlled-release coils have made an important contribution to the technique of catheter closure of coronary artery fistulas [34]. The treated vessel after occlusion becomes thrombosed to the level of first branch and the reduction in left to right shunt causes myocardial perfusion return to normal [47]. Results from the transcatheter and surgical literature show that both approaches have similar early effectiveness, morbidity, and mortality [50]. The safe and effective results of both approaches support the option for elective closure of clinically significant coronary artery fistulas in childhood [50]. Occasionally, transcatheter-based stent implantation in a restrictive coronary-pulmonary fistula is needed, to augment pulmonary flow as a palliative management option in the adult patient with complex congenital heart disease, such as pulmonary atresia, ventricular septal defect with exercise intolerance and severe cyanosis [53].

## 9. Summary

Congenital coronary artery abnormalities are rare, isolated anomalies that are important to recognize in childhood. Usually, isolated coronary anomalies are asymptomatic; however, certain forms are associated with myocardial ischemia, congestive heart failure, and sudden cardiac death in infants and children. Recognition of signs and symptoms that may indicate a congenital coronary artery anomaly should lead to additional testing, especially thorough evaluation of coronary artery anatomy using two-dimensional and color Doppler echocardiography.
